# Neonatal Renal Ultrasound Reference Values in Romanian Term Newborns: Correlations with Anthropometric Characteristics

**DOI:** 10.3390/children12091191

**Published:** 2025-09-08

**Authors:** Leonard Năstase, Adrian-Ioan Toma, Alexandru Dinulescu, Adelina Androne

**Affiliations:** 1 Neonatology Department, National Institute for Mother and Child Health “Alessandrescu-Rusescu”, 011061 Bucharest, Romania; leonard.nastase@umfcd.ro (L.N.); adelina.androne@insmc.ro (A.A.); 2 Faculty of Medicine, University of Medicine and Pharmacy “Carol Davila”, 050474 Bucharest, Romania; 3 Department of Neonatology, Life Memorial Hospital, 010719 Bucharest, Romania; adrian.toma@prof.utm.ro; 4 Faculty of Medicine, Titu Maiorescu University, 040441 Bucharest, Romania; 5 Department of Paediatrics, Emergency Hospital for Children “Grigore Alexandrescu”, 011743 Bucharest, Romania

**Keywords:** newborn, kidney, ultrasound

## Abstract

Background: The establishment of population-specific reference values for neonatal renal dimensions is essential for accurate assessment of kidney development. Currently, standardized reference data for renal volume in Romanian newborns are lacking. This study aims to establish normal renal dimensions and volumes in Romanian term newborns and evaluate their correlations with anthropometric characteristics. Methods: A prospective study was conducted at Polizu Maternity, Bucharest, Romania, involving a cohort of 100 term newborns with a gestational age (GA) between 37 and 42 weeks, all delivered at the INSMC “Alessandrescu-Rusescu” Polizu Maternity Hospital. Routine renal ultrasound was performed for all term newborns within the first 72 h of life. Renal dimensions were measured in the longitudinal and axial sections, and the volume was calculated. Results: The average kidney dimensions were as follows: length 42.0 ± 3.4 mm, width 22.6 ± 2.6 mm, and thickness 19.9 ± 2.5 mm. Renal volume ranged from 5.1 to 18.9 mL, with an average of 10.2 ± 2.5 mL. The kidney volume was significantly correlated with gestational age (r = 0.195; *p* = 0.05) and birth length (r = 0.267; *p* = 0.008), and most strongly with birth weight (r = 0.306; *p* = 0.002). Conclusions: This study provides the first reference values for renal dimensions in Romanian term newborns. Renal volume shows modest correlations with anthropometric characteristics, particularly birth weight. These reference values may serve as baseline measurements for future longitudinal studies investigating renal development and disease risk.

## 1. Introduction

Renal pathology in newborns is heterogeneous. Renal anomalies are detected prenatally in approximately 1% of fetal ultrasounds, diagnosed in less than 1% of newborns through clinical examination, and identified in 7–9% of cases during autopsy [1]. Both acquired and congenital factors contribute to renal pathology risk. Potential acquired risk factors include fetal exposure to multiple drugs, antibiotics, or other chemical substances inhaled from the environment or ingested by pregnant women through food, as well as the increasing prevalence of maternal diabetes [2,3]. Congenital factors include inherited genetic defects that affect kidney development and familial kidney diseases that are passed from parents to children [4,5]. Renal ultrasound is an effective, inexpensive, and non-invasive tool for diagnosing renal pathologies in the neonatal period [6]. Kidney size serves as a surrogate marker for nephron number, though the correlation between size and functional capacity has limitations [7,8].

Kidney size is an important predictive factor in diagnosing kidney disease and assessing intrauterine or postnatal growth. Intrauterine development primarily affects renal evolution and its associated pathologies. Maternal age, pathologies, and nutritional status during pregnancy play a key role in the structural, quantitative, and functional development of prenatal nephrons [9]. Knowing and interpreting renal dimensions is essential because they directly reflect the final number of nephrons, which physiologically completes development by 36 weeks of gestation and remains constant throughout life [10]. Prenatal renal screening only reveals obvious structural changes (e.g., polycystic kidneys, agenesis, or pelvicalyceal dilatations). However, functional impairment and reduced renal mass may occur even without prenatal findings. Risk factors for reduced renal mass or renal diseases include low birth weight, prematurity, small renal volume, or polymorphic genes [11].

The literature describes renal screening in two phases: antenatal and postnatal [12]. In Romania, neonatal abdominal ultrasound screening for renal evaluation is not yet implemented [13,14]. Consequently, renal pathologies are often diagnosed late, during the first urinary tract infection. In some renal conditions, kidney size changes before echogenicity does. The incidence of renal anomalies is growing, and many go undetected until reaching advanced stages [15]. Although some studies report neonatal renal dimensions across different populations and age groups, there are still significant differences due to racial- and demographic-specific maternal nutritional factors, which significantly impact fetal organ development [16]. Furthermore, evaluating renal health solely by length measurement is often insufficient to exclude intrauterine growth impairment [17,18]. Currently, there is no standard definition of normal renal volume in newborns, nor is there a consensus on which anthropometric parameter correlates best with kidney size [19,20].

This study aims to establish reference values for renal dimensions and volume in Romanian term newborns and determine their correlations with anthropometric characteristics. These baseline measurements may contribute to future longitudinal studies investigating renal development patterns and serve as reference standards for clinical assessment of renal growth in this population.

## 2. Materials and Methods

### 2.1. Study Design and Population

A prospective observational study was conducted at Polizu Maternity, Bucharest, Romania, involving a cohort of 100 term newborns with a gestational age (GA) between 37 and 42 weeks, all delivered at the National Institute for Maternal and Child Health “Alessandrescu-Rusescu” Polizu Maternity Hospital. Gestational age was established either based on the date of the last menstrual period or, when this was uncertain, through clinical somatic and neurological examination using the New Ballard Score.

### 2.2. Inclusion and Exclusion Criteria

Inclusion Criteria: Term newborns (37–42 weeks gestational age), physiological pregnancy evolution, and clinical stability permitting ultrasound examination within 72 h.

Exclusion Criteria: Perinatal asphyxia, known renal malformations (prenatal or postnatal), congenital anomalies affecting growth, and maternal conditions significantly impacting fetal development.

### 2.3. Ultrasound Methodology

**Figure 1:**
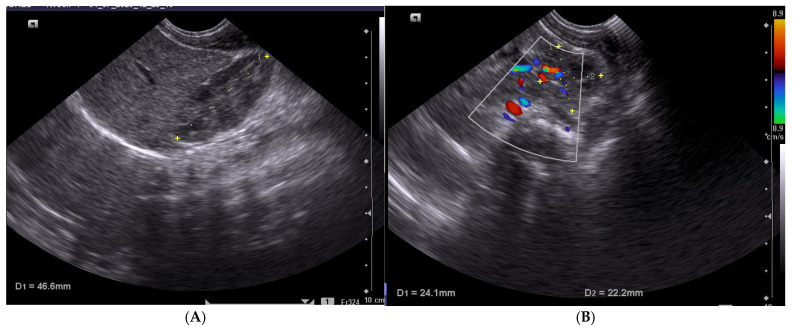
Right kidney: (**A**) longitudinal section and length measurement; (**B**) axial section with thickness (1) and width (2) measurements. (Dr. Năstase’s personal collection.)

All renal ultrasounds were performed by a single neonatologist with over 10 years of experience in neonatal abdominal ultrasonography. The ultrasound was carried out with the newborn in a supine position using a Philips Elite 7 ultrasound machine, Philips (Philips Healthcare division), Bothell, Washington (USA) and a 10 MHz microconvex probe, Philips (Philips Healthcare division), Bothell, Washington (USA) (Figure 1). To assess measurement reliability, a subset of 20 examinations was repeated by the same operator within 24 h to evaluate intra-observer variability, and intra-observer reliability was assessed using the intraclass correlation coefficient (ICC) for the subset of repeated measurements.

Renal dimensions were measured in longitudinal and axial sections. Length was defined as the distance between superior and inferior poles, width as the medio-lateral diameter, and thickness as the antero-posterior diameter. Renal volume was calculated using the ellipsoid formula: length (cm) × width (cm) × thickness (cm) × π/6. The ellipsoid formula was chosen as it is widely used in pediatric studies and provides a reasonable approximation for kidney volume, though we acknowledge limitations compared to more sophisticated methods such as 3D ultrasound or MRI volumetry.

### 2.4. Anthropometric Measurements

Birth weight was measured using calibrated electronic scales. Birth length was measured using standardized neonatal measuring boards. Given known inaccuracies in birth length measurement, particular attention was paid to proper technique and measurement standardization.

### 2.5. Statistical Analysis

Statistical analysis was performed using SPSS software version 18.0. Data were presented with 95% confidence intervals. Descriptive statistical analysis used the ANOVA test, and the distribution analysis was performed using the Kurtosis test. Pearson’s correlation coefficients were calculated for continuous variables. Analysis of covariance (ANCOVA) was performed to assess whether sex differences in renal dimensions persisted after controlling for birth weight.

### 2.6. Ethical Considerations

Written informed consent was obtained from the parents of all enrolled newborns. The study was conducted according to the Declaration of Helsinki principles and approved by the Ethics Committee of the National Institute for Maternal and Child Health (18917/27.08.2025), approved on 27 August 2025.

## 3. Results

We analyzed 106 clinically healthy term newborns with a physiological pregnancy evolution and a gestational age (GA) between 37 and 41 weeks. After excluding newborns with incidentally discovered renal anomalies, 97 patients remained eligible. The distribution of cases by gestational age was homogeneous between 37 and 41 weeks, with a mean of 38.13 ± 0.99 weeks, a median of 38 weeks, and a Kurtosis test result of 0.971. The study group consisted of 52.6% females.

Birth weight ranged from 1950 to 4300 g, with a mean of 3236 ± 397 g (Kurtosis = 0.386). Birth weight showed a weak positive correlation with gestational age (r = 0.107; *p* = 0.298), although this result cannot be generalized to the overall population. Birth length ranged from 46 to 54 cm, with a group mean of 50.95 ± 1.70 cm (Kurtosis = −0.422). Birth weight correlated strongly with birth length (r = 0.778; *p* = 0.001).

The average kidney dimensions were as follows: length 42.0 ± 3.4 mm, width 22.6 ± 2.6 mm, and thickness 19.9 ± 2.5 mm. Renal volume ranged from 5.1 to 18.9 mL, with an average of 10.2 ± 2.5 mL.

No significant differences were found between the right and left kidneys, except for length, which was significantly greater in the left kidney (42.4 mm vs. 41.7 mm; *p* = 0.035). The right kidney had a slightly higher average volume than the left (10.23 vs. 10.18 mL; *p* = 0.799).

Kidney length was significantly correlated with both birth weight and birth length (see Table 1 and Table 2). Right kidney length correlated with birth weight (r = 0.284; *p* = 0.005) and birth length (r = 0.249; *p* = 0.014). Left kidney length correlated with birth weight (r = 0.281; *p* = 0.005) and birth length (r = 0.298; *p* = 0.003).

**Figure 2:**
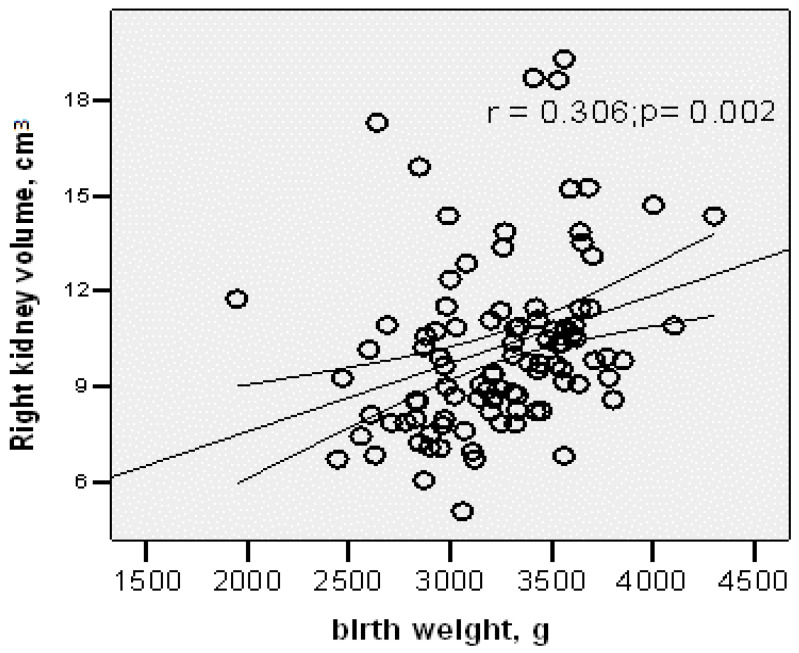
Correlation between right kidney volume and birth weight.

The right kidney volume was significantly correlated with gestational age (r = 0.195; *p* = 0.05) and birth length (r = 0.267; *p* = 0.008), and most strongly with birth weight (r = 0.306; *p* = 0.002) (Figure 2).

**Figure 3:**
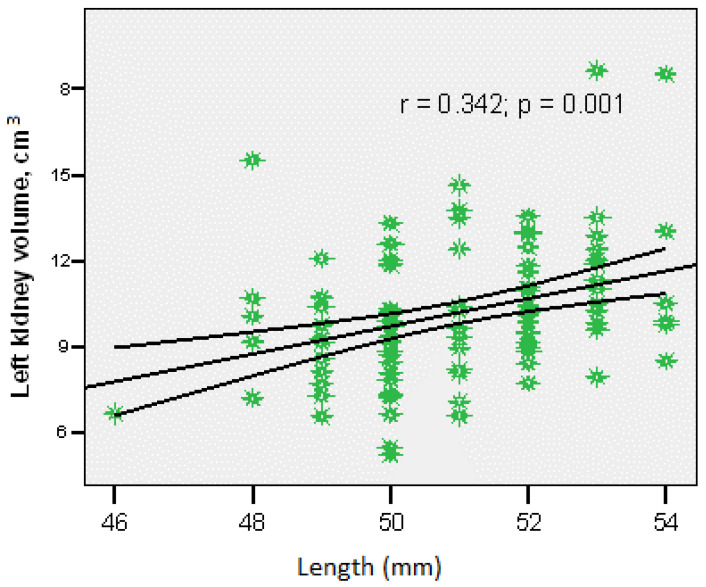
Correlation between left kidney volume and birth length.

The left kidney volume correlated most strongly with birth length (r = 0.342; *p* = 0.001) (Figure 3).

### Sex Differences

Kidney length was significantly larger in boys than in girls (right kidney 42.5 vs. 41.0 mm; *p* = 0.045 and left kidney 43.4 vs. 41.5 mm; *p* = 0.003).

**Figure 4:**
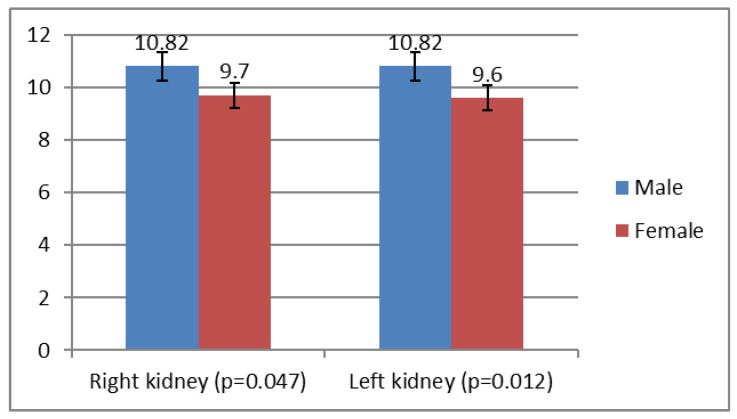
Renal volume in newborns by sex.

Renal volume was significantly higher in boys than in girls. For both kidneys, the average volume was significantly larger in males: right kidney (10.82 vs. 9.70 cm^3^; *p* = 0.047) and left kidney (10.82 vs. 9.60 cm^3^; *p* = 0.012) (Figure 4).

When analyzing the right kidney dimensions, ANCOVA analysis controlling for birth weight showed that sex differences were not significant for kidney length (*p* = 0.705), thickness (*p* = 0.471), width (*p* = 0.239), or volume (*p* = 0.791). For the case of the left kidney dimensions, the results were similar: length (*p* = 0.126), thickness (*p* = 0.880), width (*p* = 0.844), or volume (*p* = 0.102).

## 4. Discussion

Renal development begins at 9 weeks of gestation and is completed by 36 weeks. After this point, the number of nephrons remains unchanged throughout life. Renal dimensions can be affected in utero by multiple maternal, fetal, or genetic factors. There are renal pathologies that alter kidney size even before structural changes become apparent. Furthermore, physiological kidney dimensions vary by age across different populations depending on demographic and ethnic characteristics [21]. Therefore, understanding the renal dimensions of term newborns at birth is extremely important, as these can serve as a reference for assessing subsequent kidney development and predicting the risk of renal diseases.

This study aims to identify normal neonatal renal values in the Romanian population and determine which anthropometric parameters are most strongly correlated with renal size and volume. As a general rule, normal kidney length is considered to be approximately equal (in mm) to gestational age in weeks [22]. Enlarged kidneys may be associated with hydronephrosis, multicystic dysplasia, polycystic kidney disease, or congenital nephrotic syndrome. Small kidneys suggest renal dysplasia or hypoplasia [23,24].

Our study demonstrates that renal volume correlates with both gestational age and birth weight. This suggests that impaired fetal perfusion leading to small for gestational age (SGA) or intrauterine growth restriction (IUGR) also results in smaller kidney dimensions. The purpose of the study was to determine which kidney measurement is most useful for evaluating normal renal development at the time of examination. Renal ultrasound is non-invasive, inexpensive, and very useful for identifying renal pathology that allows for treatment and follow-up.

In our cohort of term newborns, average renal dimensions were as follows:

**Length**: 42.0 ± 3.4 mm;

**Width**: 22.6 ± 2.6 mm;

**Thickness**: 19.9 ± 2.5 mm;

**Renal volume**: ranged from 5.1 to 18.9 mL, with a mean of 10.2 ± 2.5 mL.

Normal values in term newborns are reported in only a few international studies, often with small sample sizes [25,26,27,28]. Modern ultrasound technology offers better resolution and improved measurement accuracy. Most existing studies focus only on kidney length. The values obtained in the current study are similar to those reported in Caucasian populations and higher than those in Asian populations. Until now, no studies have been conducted in the Romanian population.

Significant differences were found between the two kidneys in terms of length, with the left kidney being longer than the right (42.4 mm vs. 41.7 mm; *p* = 0.035). This difference has also been described in infants and older children, where the left kidney is typically 2–4 mm longer [25,26,27,28]. Only one study on newborns found no significant difference between the two kidneys on ultrasound [29]. In preterm infants, no differences in kidney dimensions are reported [30].

There were significant sex-related differences in both kidney length and volume. The left and right kidneys were larger in boys than in girls by approximately 2 mm (43.4 vs. 41.5 mm; *p* = 0.003) and 1.5 mm (42.5 vs. 41.0 mm; *p* = 0.045), respectively. Renal volume was also higher in boys, with an average difference of about 1.2 mL (10.82 vs. 9.60 cm^3^; *p* = 0.012). When controlling for birth weight using ANCOVA analysis, the sex-related differences in renal dimensions were no longer statistically significant, suggesting that observed differences are primarily attributed to birth weight variations rather than intrinsic sex-related developmental factors. Studies on preterm infants report similar sex differences, although studies in infants and older children do not consistently find significant differences in kidney size when assessed radiologically or sonographically [25,29,31].

In newborns and infants, most studies focus only on renal length, while renal volume is less frequently assessed.

Several renal pathologies can affect kidney size. Small kidneys are associated with renal hypoplasia, dysplasia, multicystic kidney, and other conditions [23]. Renal hypoplasia may be caused by multiple genetic mutations (e.g., HFN1B, PAX2, PXB1) as well as environmental factors such as intrauterine growth restriction (IUGR), maternal diabetes, hypertension, anti-inflammatory or antihypertensive drug use during pregnancy, or maternal alcohol and tobacco consumption [31,32]. Renal impairment can be diagnosed antenatally or postnatally when kidney volume is more than two standard deviations below the mean [33]. Conversely, increased kidney size may result from compensatory hypertrophy due to contralateral renal dysfunction, high protein intake, diuretic use, or other pathologies [34].

Kidney length was correlated with both birth weight and birth length for both kidneys. Right kidney volume showed significant correlation with gestational age (r = 0.195; *p* = 0.05), birth weight (r = 0.306; *p* = 0.002), and birth length (r = 0.267; *p* = 0.008). Left kidney volume showed the strongest correlation with birth length (r = 0.342; *p* = 0.001). The most robust correlations were between right kidney volume and birth weight, and between left kidney volume and birth length.

Other authors have also reported a strong correlation between birth weight and both kidney length and volume in studies conducted on preterm infants [30].

Since birth weight and length correlate with renal volume, it is important to assess kidneys postnatally in term newborns with birth weight below the 10th percentile or a ponderal index below 10%. Our findings provide baseline reference data for Romanian term newborns that may inform clinical practice. However, the clinical utility of these measurements for predicting renal disease risk requires validation through longitudinal studies tracking renal function and disease development over time.

Future research should include:

1. Larger, multicenter studies to establish more robust reference ranges;
2. Longitudinal follow-up to assess predictive value for renal outcomes;
3. Inclusion of maternal factors (hypertension, diabetes, medication use);
4. Comparison with other populations to understand ethnic and demographic variations;
5. Investigation of preterm infants, who may have different risk profiles.

Population-Specific Considerations

This study represents the first systematic evaluation of renal dimensions in Romanian newborns. While our values align with other European populations and differ from some Asian cohorts, establishing population-specific references remains important given known ethnic and demographic influences on organ development.

The lack of systematic neonatal renal screening in Romania creates opportunities for future implementation, though cost-effectiveness and clinical utility require careful evaluation before widespread adoption [13]. Our study is the first renal ultrasound reference value study in the Romanian neonatal population. It provides valuable data on renal length, width, thickness, and volume, which may contribute alongside future, larger studies to establish reference nomograms for renal dimensions in Romanian term newborns.

### Study Limitations

This study has several important limitations that must be acknowledged. The sample size of 97 newborns, while adequate for initial reference value establishment, is relatively small for comprehensive population standards. Larger, multicenter studies would provide more robust reference ranges and better account for regional variations within Romania.

The correlations observed between renal volume and anthropometric parameters, while statistically significant, are modest in strength (r = 0.195–0.342). These correlations explain only 4–12% of the variance in renal volume, indicating that other unmeasured factors significantly influence kidney size. The presence of outliers in our correlation plots further emphasizes the individual variability in renal development.

Kidney size serves as an imperfect surrogate for functional capacity. While larger kidneys generally contain more nephrons, the relationship between size and function is complex and influenced by factors such as glomerular density, tubular development, and vascular architecture. Our study cannot directly assess the functional implications of size variations.

We measured renal volume only in the first 72 h of life without longitudinal follow-up. The clinical relevance of early size measurements for predicting long-term outcomes remains to be established through prospective studies with extended follow-up periods.

Maternal factors known to influence renal development (hypertension, diabetes, medications, nutritional status) were not systematically analyzed, limiting our ability to account for these potential confounding variables.

Technical considerations: Renal volume calculation using the ellipsoid formula provides a reasonable approximation but has known limitations [35]. More sophisticated methods, such as 3D ultrasound reconstruction or MRI volumetry, offer superior accuracy but are not routinely available in neonatal settings [36,37]. Other formulas suggested in the literature include the prolate ellipsoid formula, Cavalieri principle-based calculations, and 3D reconstruction methods. Each approach has inherent limitations—the ellipsoid formula may overestimate volume in irregularly shaped kidneys, while more complex methods require specialized equipment not routinely available in neonatal settings [38]. Future studies might compare different measurement techniques to validate our approach.

The single-operator measurement approach, while ensuring consistency, limits generalizability to broader clinical practice. Assessment of inter-observer variability would strengthen the practical applicability of these reference values.

## 5. Conclusions

This study establishes preliminary reference values for renal dimensions and volume in Romanian term newborns. Renal volume shows modest correlations with anthropometric characteristics, particularly birth weight. These findings provide baseline measurements for future research but require validation through larger studies with longitudinal follow-up to establish clinical utility. The reference values presented here represent a first step toward understanding normal renal development in this population. However, the relationship between neonatal kidney size and long-term renal health remains to be definitively established. Future longitudinal studies are needed to determine whether early ultrasound measurements can effectively identify newborns at risk for chronic kidney disease.

## Figures and Tables

**Table 1:** Correlation between kidney dimensions and birth weight.

Birth Weight	<2800 g	2800–3500 g	>3500 g	F_ANOVA_ test *p*	<2800 g	2800–3500 g	>3500 g	F_ANOVA_ test *p*
Dimension	Right kidney				Left kidney			
Length (mm)	40.8 ± 2.2	41.25 ± 4.2	43.00 ± 2.7	0.085	41.37 ± 2.3	41.97 ± 3.4	43.72 ± 2.6	0.026
Thickness (mm)	19.2 ± 2.8	19.63 ± 2.4	21.42 ± 2.5	0.004	19.31 ± 3.1	19.97 ± 2.1	21.34 ± 2.7	0.016
Width (mm)	22.5 ± 3.3	22.56 ± 2.3	23.89 ± 2.9	0.080	21.92 ± 3.0	22.20 ± 2.1	23.12 ± 3.0	0.230
Volume (mL)	9.47 ± 3.0	9.65 ± 2.4	11.65 ± 2.9	0.004	9.26 ± 2.5	9.77 ± 2.13	11.32 ± 2.5	0.006

**Table 2:** Correlation between kidney dimensions and birth length.

Dimensions of kidney	<50 cm	>50 cm	*p*	<50 cm	>50 cm	*p*
	Right kidney			Left kidney		
Length (mm)	40.60 ± 3.45	42.72 ± 3.68	0.004	41.46 ± 3.18	43.25 ± 2.98	0.005
Thickness (mm)	19.46 ± 2.57	20.70 ± 2.51	0.018	19.47 ± 2.24	20.91 ± 2.63	0.005
Width (mm)	22.64 ± 2.72	23.23 ± 2.63	0.287	21.96 ± 2.44	22.86 ± 2.69	0.089
Volume (mL)	9.48 ± 2.54	10.88 ± 2.84	0.013	9.36 ± 2.13	10.88 ± 2.43	0.002

## Data Availability

The original contributions presented in the study are included in the article; further inquiries can be directed to the corresponding author.

## Abbreviations

The following abbreviations are used in this manuscript:

GA	Gestational age
ANOVA	Statistical test
IUGR	Intrauterine growth restriction
